# A Predictive Model for Post-Percutaneous Coronary Intervention Readmission: Insights from Machine Learning on Clinical Risk Factors

**DOI:** 10.34172/jrhs.13875

**Published:** 2026-08-15

**Authors:** Maryam Farhadian, Seyed Kianoosh Hosseini, Tahereh Roostami

**Affiliations:** ^1^Research Center for Health Sciences, Institute of Health Sciences and Technologies, Avicenna Health Research Institute, Hamadan University of Medical Sciences, Hamadan, Iran; ^2^Department of Biostatistics, School of Public Health, Hamadan University of Medical Sciences, Hamadan, Iran; ^3^Department of Cardiology, School of Medicine, Farshchian Cardiovascular Subspecialty Medical Center, Hamadan University of Medical Sciences, Hamadan, Iran

**Keywords:** Myocardial infarction, Patient readmission, Percutaneous coronary intervention, Machine learning, Risk assessment

## Abstract

**Introduction:**

Unplanned one-year readmission after percutaneous coronary intervention (PCI) for ST-segment elevation myocardial infarction (STEMI) poses serious clinical and economic challenges. This study developed and validated a random forest (RF) model to predict one-year all-cause unplanned readmission in STEMI-PCI patients and identify key risk factors.

**Study Design:**

A single-center retrospective cohort study.

**Methods:**

This single-center retrospective cohort study was conducted at Farshchian Heart Hospital from September 2021 to September 2024. Overall, 1,836 STEMI patients undergoing primary PCI were analyzed to predict 365-day unplanned readmission. The primary outcome was 365-day unplanned readmission (binary: readmitted or not). A RF classifier was developed on an 80/20 training-test split, optimized through repeated cross-validation with SMOTE for class imbalance. A SMOTE-RF model was also employed to mitigate the significant class imbalance inherent in the 17.6% readmission rate. Finally, key predictors were identified using mean decrease in Gini impurity.

**Results:**

Operating at an optimized threshold, the RF model demonstrated moderate discriminative ability (AUC: 0.765) with a sensitivity of 62.5% and specificity of 78.5%. Moreover, the RF model outperformed logistic regression in terms of AUC (0.695 vs. 0.765) and specificity (78.5% vs. 52%), although logistic regression achieved higher sensitivity (78.5% vs. 62.5%). Multivessel disease, smoking, length of stay, hypertension, ejection fraction, and age were key predictors of readmission identified by the RF.

**Conclusion:**

The RF model exhibited acceptable predictive performance for one-year hospital readmission, enabling clinically interpretable risk stratification and identifying key clinical risk factors to inform tailored post-discharge care. However, external validation is required to confirm its generalizability and clinical applicability.

## Introduction

 ST-segment elevation myocardial infarction (STEMI) remains a critical cardiovascular emergency, with significant advances in acute reperfusion therapy leading to decreased in-hospital mortality.^1-3^ In this regard, healthcare quality assessment has increasingly emphasized long-term patient outcomes, particularly unplanned hospital readmissions within one-year post-discharge. Such readmissions serve as the critical indicators of disruptions in the post-acute care continuum, frequently reflecting deficiencies in hospital care management, discharge planning, patient education, or transitional care coordination. Beyond their clinical implications, these readmissions impose substantial economic burdens on healthcare systems while also adversely affecting patients’ quality of life and functional recovery.^4-8^

 Post-STEMI readmissions are multifactorial, arising from both cardiac complications (e.g., heart failure, recurrent ischemia, arrhythmias, or stent-related issues) and non-cardiac factors, including exacerbation of comorbidities (e.g., diabetes, chronic kidney disease, and pulmonary conditions), medication non-adherence, and socioeconomic barriers to care. According to recent studies, 20–30% of these readmissions are potentially preventable through enhanced care coordination, patient education, and targeted post-discharge interventions, highlighting the critical need for precise risk stratification tools to identify high-risk patients prior to discharge.^9-13^

 Current approaches for predicting readmission risk following STEMI-percutaneous coronary intervention (PCI) are largely based on conventional statistical models, such as logistic regression and Cox proportional hazard analysis.^14-16^ While these models offer interpretability and clinical familiarity, they require pre-specified interactions and assume linear predictor-outcome relationships. Consequently, such models are limited in capturing the complex, non-linear interactions among the multifactorial determinants of readmission risk, which encompass demographic, clinical, procedural, laboratory, and psychosocial domains.^15-17^

 The integration of machine learning, particularly ensemble methods, such as random forest (RF), represents a significant paradigm shift in clinical prediction by enabling the automatic detection of complex data patterns without explicit model specification. RF mitigates overfitting and maintains robustness against noise and correlated predictors, which are common challenges in electronic health record data. However, the application of this method is accompanied by limitations, including the need for larger sample sizes and the risk of overfitting without rigorous hyperparameter tuning. Moreover, while offering feature importance metrics, RF lacks the coefficient-based transparency of traditional models (e.g., logistic regression), potentially constraining interpretability. Nonetheless, RF achieves a favorable balance between predictive accuracy and interpretability relative to more opaque models, rendering it well-suited for clinical contexts that demand reliable performance and actionable insight.^18-25^

 Although machine-learning applications are increasingly being used in cardiovascular care, few studies have specifically focused on one-year unplanned readmission within a dedicated STEMI-PCI cohort. Notably, existing models often rely on limited predictor variables, employ shorter follow-up durations (e.g., 30 days), or target broader acute coronary syndrome populations, and they frequently lack rigorous internal validation with appropriate handling of class imbalance—an important consideration given the typically low readmission rates in these groups.^26-29^

 To our knowledge, no peer-reviewed studies have so far specifically reported readmission rates for this condition in Hamadan province. However, a recent Iranian study conducted in Isfahan (773 STEMI patients) reported a 3-year readmission rate of 30.27%.^4^

 Accordingly, this study aims to develop and internally validate a RF model to predict one-year unplanned hospital readmission among STEMI patients undergoing PCI, utilizing a comprehensive set of predictors. By applying an interpretability framework, the research will identify and rank the key determinants of readmission risk, thereby informing the design of targeted intervention strategies. The predictive accuracy of the model is subsequently benchmarked against conventional logistic regression.

## Methods

###  Study Design and Population

 This single-center retrospective cohort study was performed at Farshchian Heart Hospital in Hamadan, Iran, and included all consecutive adult patients ( ≥ 18 years) diagnosed with STEMI between September 2021 and September 2024. Cases were identified using the International Classification of Disease (tenth revision) codes (I21.0, I21.1, I21.2, and I21.9) and confirmed based on the guidelines of the American Heart Association/American College of Cardiology and European Society of Cardiology. The inclusion criterion required treatment with primary PCI within 12 hours of symptom onset, While the exclusion criteria included in-hospital mortality, elective PCI, coronary artery bypass grafting during admission, death within 24 hours, and incomplete medical records.

###  Outcome Definition

 The primary outcome was 365-day unplanned hospital readmission, which was confirmed through institutional records and validated by chart review. Additionally, patients who died during index hospitalization or within the one-year follow-up period were excluded from the analysis in order to account for the competing risk of death.

###  Data Collection and Variable Selection

 Baseline demographic, clinical, procedural, and laboratory data were extracted from the electronic health record system. Variables included age, gender, comorbidities (hypertension, diabetes, chronic kidney disease, chronic obstructive pulmonary disease, and smoking status), and procedural details (PCI type, target vessel, number of diseased vessels, stents placed, and access site). Other variables were vital signs at admission, echocardiographic ejection fraction (EF), and comprehensive laboratory panels (cardiac enzymes, lipid profile, renal function, coagulation, and complete blood count). Likewise, continuous variables were recorded as measured, and categorical variables were defined per institutional guidelines. It should be noted that all 42 clinically relevant variables were included in the RF model, which automatically selects important features during training. Final interpretation focused only on the top-ranked predictors.

###  Statistical Analysis

 Continuous variables were reported as means ± standard deviations and compared using Student’s t-tests or Mann–Whitney U tests as appropriate. In addition, categorical variables were presented as frequencies (%) and underwent comparison using chi-square or Fisher’s exact tests. Further, a two-sided *P*-value < 0.05 was considered statistically significant. Finally, analyses were performed using R version 4.3.0 (R Foundation for Statistical Computing).

 To benchmark the performance of the RF model, a conventional multivariable logistic regression model was developed using the same training dataset and predictor variables. Then, all models underwent identical cross-validation tuning and synthetic minority over-sampling technique (SMOTE) preprocessing.

###  Data Preprocessing and Splitting

 Data preprocessing followed a multi-step protocol to enhance dataset quality. First, records with irrelevant or duplicate entries were removed, and variables with a low missing value ( < 5%) underwent imputation using variable-specific central tendency metrics (means and modes for continuous and categorical variables, respectively). Furthermore, features exhibiting a higher missing value ( > 5%) were imputed via k-nearest neighbors (k = 5).

 The dataset was randomly partitioned into training (80%, n = 1,470) and hold-out test (20%, n = 366) sets, with stratification on readmission status to preserve outcome distribution.

###  Random Forest Modeling

 The RF method is an ensemble learning technique that builds and trains numerous decision trees and then outputs the most common prediction result from these trees when used for classification. Moreover, the algorithm is implemented using the RF package in R. The model functions through:

 Likewise, the process involves creating bootstrap samples from the training data, constructing unpruned, deep trees on each sample, selecting a random subset of predictors at each split (mtry), and then combining the predictions across all trees through majority voting.^30^

###  Handling Class Imbalance

 Four strategies were assessed using RF classification to address the imbalanced outcome (17.6% readmission rate), including (1) no adjustment (original RF), (2) class weighting (RF balanced), (3) down-sampling the majority class (RF down), and (4) applying the synthetic minority oversampling technique to the training set.

 To address class imbalance, the SMOTE was exclusively applied to the training set after data splitting. Specifically, following the 80/20 stratified split, the training data (n = 1,470) underwent SMOTE to generate synthetic instances of the minority class (readmitted patients). Nonetheless, the test set (n = 366) remained untouched and reflected the original class distribution (17.6% readmission) to ensure unbiased performance evaluation. This sequential approach, split then SMOTE, rigorously prevented data leakage, as no information from the test set influenced synthetic sample generation or model training.

###  Model Training and Hyper Parameter Tuning

 Hyper parameters were optimized via repeated (3 × ) 5-fold cross-validation on the training set. The tuned hyper parameters were as follows:

- *Number of Variables Randomly Sampled at Each Split (mtry):* 2, 3, 4, 5, 6, 8, 10
- *Number of Trees (ntree):* 100, 200, 300, 500, 1000
- *Maximum Tree Depth (max_depth):* 10, 20, 30, unlimited
- *Minimum Node Size (min_node_size):* 1, 3, 5, 10
- *Node Splitting Criterion:* Gini impurity (default)

The final optimized parameters were: mtry = 5, ntree = 300, max_depth = unlimited, min_node_size = 1. Unlimited depth and minimum node size of 1 were selected to allow trees to grow fully, capturing complex interactions while relying on ensemble averaging to control overfitting. Moreover, cross-validation area under the curve (AUC) was the primary optimization metric.

###  Model Evaluation

 Performance was assessed on the independent test set using area under the receiver operating characteristic curve (AUC), sensitivity, specificity, accuracy, positive predictive value (PPV), and negative predictive value (NPV). Additionally, the optimal probability threshold was determined using AUC and Youden’s J statistic. Likewise, model calibration was evaluated across risk deciles. Eventually, clinical utility was analyzed by applying a 3:1 cost ratio (false negative: false positive), reflecting the higher clinical consequence of missing a readmission prediction.

###  Model Interpretation

 To interpret the model, the mean decrease in impurity, based on Gini impurity criterion, was used to rank the importance of predictor variables.

 In addition, the final model was employed to stratify patients into five risk categories (very low to very high) based on predicted probability cut points, and the observed readmission rates were calculated for each category.

## Results

 A total of 1,836 post-PCI STEMI patients were included in the final analysis. Of these, 324 (17.6%) were readmitted within one year, while 1,512 (82.4%) were not readmitted. Complete baseline characteristics of the cohort, stratified by readmission status, are presented in Table 1.

**Table 1:** Comparison of Categorical Baseline Characteristics Among Post-PCI STEMI Patients, Stratified by 1-Year Readmission Status

**Variables**	**Not Readmitted, n=1512 (82.4%)**		**Readmitted,** **n=324 (17.6%)**		***P*** **-Value**
	**Number**	**%**	**Number**	**%**	
Gender					0.096
Female	306	79.5	79	20.5	
Male	1206	83.1	245	16.9	
Target vessel					0.017
Left anterior descending	847	84.4	156	15.6	
Left circumflex	151	79.5	39	20.5	
Right coronary artery	445	78.9	119	21.1	
Multi-vessel	69	87.3	10	12.7	
Number of vessels (vessel score)					0.001
1	817	90.3	88	9.7	
2	484	78.1	136	21.9	
3	211	67.8	100	32.2	
Number of stents					0.001
0	36	61.0	23	39.0	
1	1237	83.4	246	16.6	
2 +	239	81.3	55	18.7	
Vascular access					0.847
Radial	1237	82.3	297	17.7	
Femoral	239	82.9	27	17.1	
Hypertension					0.001
Positive	824	79.1	218	20.9	
Negative	673	84.8	121	15.2	
Diabetes					0.004
Positive	1184	83.7	230	16.3	
Negative	328	77.7	94	22.3	
History of heart disease					0.002
Positive	1164	84.2	219	15.8	
Negative	290	76.9	87	23.1	
Unknown/none	58	76.3	18	23.7	
Family history of premature CAD					0.074
Positive	328	77.7	94	22.3	
Negative	1249	83.1	254	16.9	
Unknown/none	263	79.0	70	21.0	
Smoking					0.001
Yes	760	85.8	126	14.2	
No	694	78.9	186	21.1	
Unknown/none	58	82.9	12	17.1	
Chronic kidney disease					0.017
Positive	1487	82.7	312	17.3	
Negative	25	67.6	12	32.4	
Chronic obstructive pulmonary Disease					0.002
Positive	1482	82.8	308	17.2	
Negative	30	65.2	16	34.8	
Troponins					0.001
Positive	922	86.1	149	13.9	
Negative	590	77.1	175	22.9	

*Note*. CAD: Cardiovascular disease; RE: Random forest; SMOTE: Synthetic minority over-sampling technique.

 The number of diseased vessels demonstrated a dose-response relationship with readmission, increasing from 9.7% for single-vessel disease to 32.2% for triple-vessel disease (*P* < 0.001). Further, target vessel involvement significantly differed (*P* = 0.017), with higher rates observed for right coronary artery (21.1%) and left circumflex artery (20.5%) than in left anterior descending artery (15.6%). Patients who received no stent placement experienced markedly higher readmission rates (39.0% vs. with stent, *P* < 0.001). Gender and vascular access site showed no significant associations.

###  Comorbidities and Risk Factors

 Readmitted patients had a higher prevalence of several conditions, including hypertension (20.9% vs. 15.2%, *P*= 0.001), diabetes mellitus (22.3% vs. 16.3%, *P* = 0.004), smoking (21.1% vs. 14.2%, *P*= 0.001), chronic kidney disease (32.4% vs. 17.3%, *P* = 0.017), chronic obstructive pulmonary disease (34.8% vs. 17.2%, *P*= 0.002), and history of heart disease (23.1% vs. 15.8%, *P*= 0.002). Notably, patients with negative troponin at admission were readmitted more frequently than those with positive troponin (22.9% and 13.9%, respectively, *P*< 0.001, Table 1).

###  Clinical and Laboratory Parameters

 Readmitted patients were significantly older (62.88 ± 9.34 vs. 60.78 ± 10.66 years, *P*< 0.001) and had longer initial hospital stays (3.54 ± 2.07 vs. 3.22 ± 1.78 days, *P*= 0.005). Furthermore, they exhibited higher systolic blood pressure (142.62 ± 27.71 vs. 138.26 ± 26.41 mmHg, *P*= 0.008) but lower EF (38.05% ± 8.86 vs. 39.82% ± 8.51, *P*= 0.001). The results of laboratory tests (Table 2) revealed higher concentrations of glucose (177.25 ± 100.85 vs. 164.68 ± 88.31 mg/dL, *P*= 0.024), triglycerides (161.35 ± 90.14 vs. 150.22 ± 88.81 mg/dL, *P*= 0.041), and LDL cholesterol (98.28 ± 47.58 vs. 92.68 ± 43.75 mg/dL, *P*= 0.038), as well as higher levels of serum creatinine (1.07 ± 0.41 vs. 1.02 ± 0.26, *P*= 0.02) and uric acid (6.21 ± 3.09 vs. 5.78 ± 2.12, *P*= 0.002).

**Table 2:** Baseline Characteristics and Clinical Parameters of Post-PCI STEMI Patients, Stratified by 1-Year Readmission Status

**Variables**	**Not Readmitted**		**Readmitted**		**Mean Difference (95% CI)**	***P*** **-Value**
	**Mean**	**SD**	**Mean**	**SD**		
Age (year)	60.78	10.66	62.88	9.34	2.10 (0.85 to 3.35)	0.001
Length of stay (1st admission day)	3.22	1.78	3.54	2.07	0.32 (0.10 to 0.54)	0.005
Vital signs and hemodynamics						
Systolic blood pressure (mmHg)	138.26	26.41	142.62	27.71	4.36 (1.16 to 7.56)	0.008
Diastolic blood pressure (mmHg)	87.99	25.39	89.18	20.54	1.19 (-1.77 to 4.14)	0.431
Pulse rate (bpm)	80.22	16.52	81.37	15.16	1.15 (-0.81 to 3.10)	0.25
Ejection fraction (%)	39.82	8.51	38.05	8.86	-1.78 (-2.81 to -0.75)	0.001
Coagulation profile						
Prothrombin time (sec)	13.46	1.75	13.44	1.89	-0.02 (-0.24 to 0.19)	0.838
International normalized ratio	1.12	0.32	1.12	0.25	0.00 (-0.04 to 0.04)	0.925
Partial thromboplastin time (sec)	31.33	14.07	31.84	15.27	0.51 (-1.21 to 2.23)	0.559
Renal and metabolic						
Blood glucose (mg/dL)	164.68	88.31	177.25	100.85	12.57 (1.69 to 23.45)	0.024
Fasting glucose (mg/dL)	137.69	57.12	140.70	57.07	3.01 (-3.85 to 9.87)	0.389
BUN (mg/dL)	17.29	7.93	17.96	7.10	0.67 (-0.27 to 1.60)	0.163
Creatinine (mg/dL)	1.02	0.26	1.07	0.41	0.04 (0.01 to 0.08)	0.02
Sodium (mEq/L)	139.43	3.22	139.09	3.16	-0.34 (-0.72 to 0.05)	0.084
Potassium (mEq/L)	4.14	0.43	4.21	0.76	0.07 (0.01 to 0.13)^†^	0.111
Uric acid (mg/dL)	5.78	2.12	6.21	3.09	0.44 (0.16 to 0.71)	0.002
Cardiac biomarkers and lipids						
Total cholesterol (mg/dL)	163.19	50.69	167.87	50.71	4.69 (-1.40 to 10.77)	0.131
Triglycerides (mg/dL)	150.22	88.81	161.35	90.14	11.13 (0.44 to 21.82)	0.041
HDL cholesterol (mg/dL)	40.53	12.80	41.12	10.52	0.59 (-0.91 to 2.08)	0.441
LDL cholesterol (mg/dL)	92.68	43.75	98.28	47.58	5.60 (0.27 to 10.94)	0.038
Complete blood count						
White blood cells ( × 10³/µL)	10.07	3.26	10.43	3.99	0.36 (-0.05 to 0.77)	0.084
Red blood cells ( × 10⁶/µL)	4.97	1.32	4.98	0.52	0.01 (-0.13 to 0.16)	0.856
Hemoglobin (g/dL)	14.79	2.23	14.65	1.80	-0.13 (-0.39 to 0.13)	0.316
Hematocrit (%)	42.92	4.86	43.05	4.65	0.13 (-0.45 to 0.71)	0.667
Red cell distribution width (%)	13.42	1.41	13.28	0.97	-0.14 (-0.30 to 0.02)	0.087
Platelets ( × 10³/µL)	216.41	66.63	216.04	64.23	-0.37 (-8.32 to 7.58)	0.927
MCV (fL)	87.02	6.81	86.66	5.21	-0.36 (-1.15 to 0.43)	0.369
MCH (pg)	29.96	2.86	29.83	2.00	-0.13 (-0.46 to 0.20)	0.432
MCHC (g/dL)	34.36	3.50	34.34	1.46	-0.02 (-0.41 to 0.37)	0.918

*Note*. PCI: Percutaneous coronary intervention; STEMI: ST-segment elevation myocardial infarction; SD: Standard deviation; CI: Confidence interval; BUN: Blood urea nitrogen; HDL: High-density lipoprotein; LDL: Low-density lipoprotein; MCV: Mean corpuscular volume; MCH: Mean corpuscular hemoglobin; MCHC: Mean corpuscular hemoglobin concentration.

###  Data Preparation and Model Selection

 The dataset was randomly split into training (n = 1,470, 80%) and hold-out test (n = 366, 20%) sets, with stratification to preserve the readmission ratio. First, multiple class imbalance handling strategies were evaluated for a RF classifier. Based on the results (Table 2), the unadjusted model demonstrated high sensitivity (0.844) but poor specificity (0.500), reflecting a significant bias toward the majority class. Alternative approaches improved specificity but substantially compromised sensitivity. Ultimately, the SMOTE-based model achieved an optimal balance, reaching the highest accuracy of 0.757 while maintaining reasonable sensitivity of 0.625 and specificity of 0.785 (Table 3).

**Table 3:** Performance Comparison of Random Forest Models With Different Class Imbalance Handling Strategies

**Model**	**Sensitivity**	**Specificity**	**Accuracy**	**AUC**	**Threshold**
Logistic regression + SMOTE	0.785	0.520	0.612	0.695	0.391
Original RF	0.844	0.500	0.560	0.713	0.165
RF balanced	0.578	0.755	0.724	0.685	0.441
RF down sampling	0.734	0.669	0.680	0.752	0.409
RF + SMOTE	0.625	0.785	0.757	0.765	**0.255**

*Note*. SMORE: Synthetic minority over-sampling technique.; RF: Random forest; AUC: Area under the curve.

###  Final Model Performance 

 The final SMOTE-based RF model, tuned via repeated (3x) 5-fold cross-validation (optimal mtry = 5, 300 trees), displayed good discriminatory ability on the independent test set (AUC = 0.765). Using a probability threshold of 0.255 (optimized via AUC), the model achieved a sensitivity of 62.5%, a specificity of 78.5%, and an accuracy of 75.7%. Moreover, the PPV was 38.1%, whereas the NPV reached 90.8%. The confusion matrix is illustrated in Figure 1.

**Figure 1:**
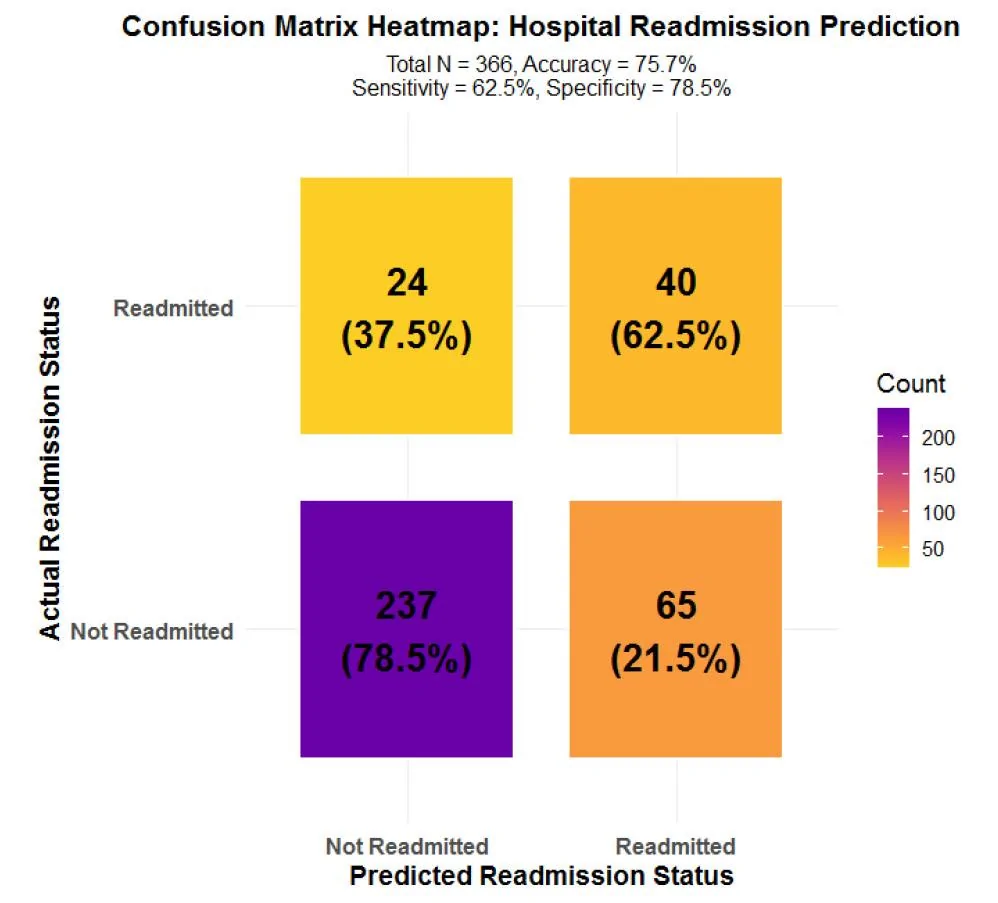
Confusion Matrixes for Test Data Based on theTuned RF-SMOTE Model. *Note*. RE: Random forest; SMOTE: Synthetic minority over-sampling technique

 In contrast, the SMOTE-based logistic regression model, with an optimized threshold of 0.391, achieved 78.5% sensitivity, 52% specificity, and 61.2% accuracy.

###  Feature Importance and Model Interpretation

 Variable importance analysis based on the Gini index identified the top predictors of readmission as: the presence of multiple vessel disease, initial length of stay (LOS), smoking status, age, EF, and hypertension (Figure 2).

**Figure 2:**
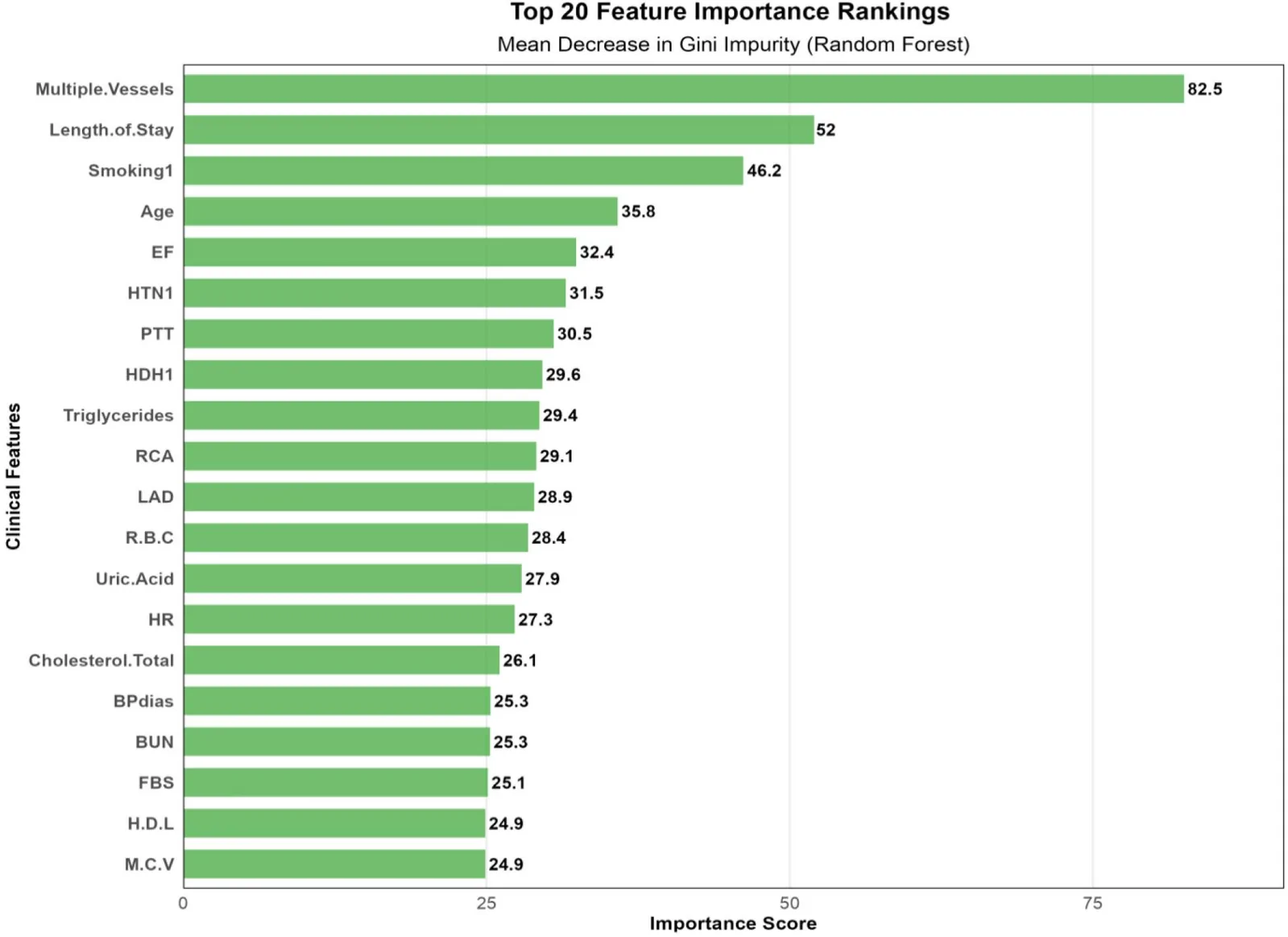
Feature Importance (Gini) Bar Chart Based on the Tuned RF-SMOTE Model (Top 20 Features). *Note*. RE: Random forest; SMOTE: Synthetic minority over-sampling technique

###  Model Calibration and Clinical Utility

 The model showed reasonable calibration across risk deciles (Figure 3). A clinical utility analysis with a 3:1 cost ratio for false negatives versus false positives reflects the greater clinical impact of missing readmission to minimize weighted costs. The final model (threshold = 0.255) categorized test patients into five risk groups with steadily rising observed readmission rates: very low (5.8%), low (8.3%), medium (29.4%), high (60.0%), and very high (100.0%), as evidenced by effective risk discrimination.

**Figure 3:**
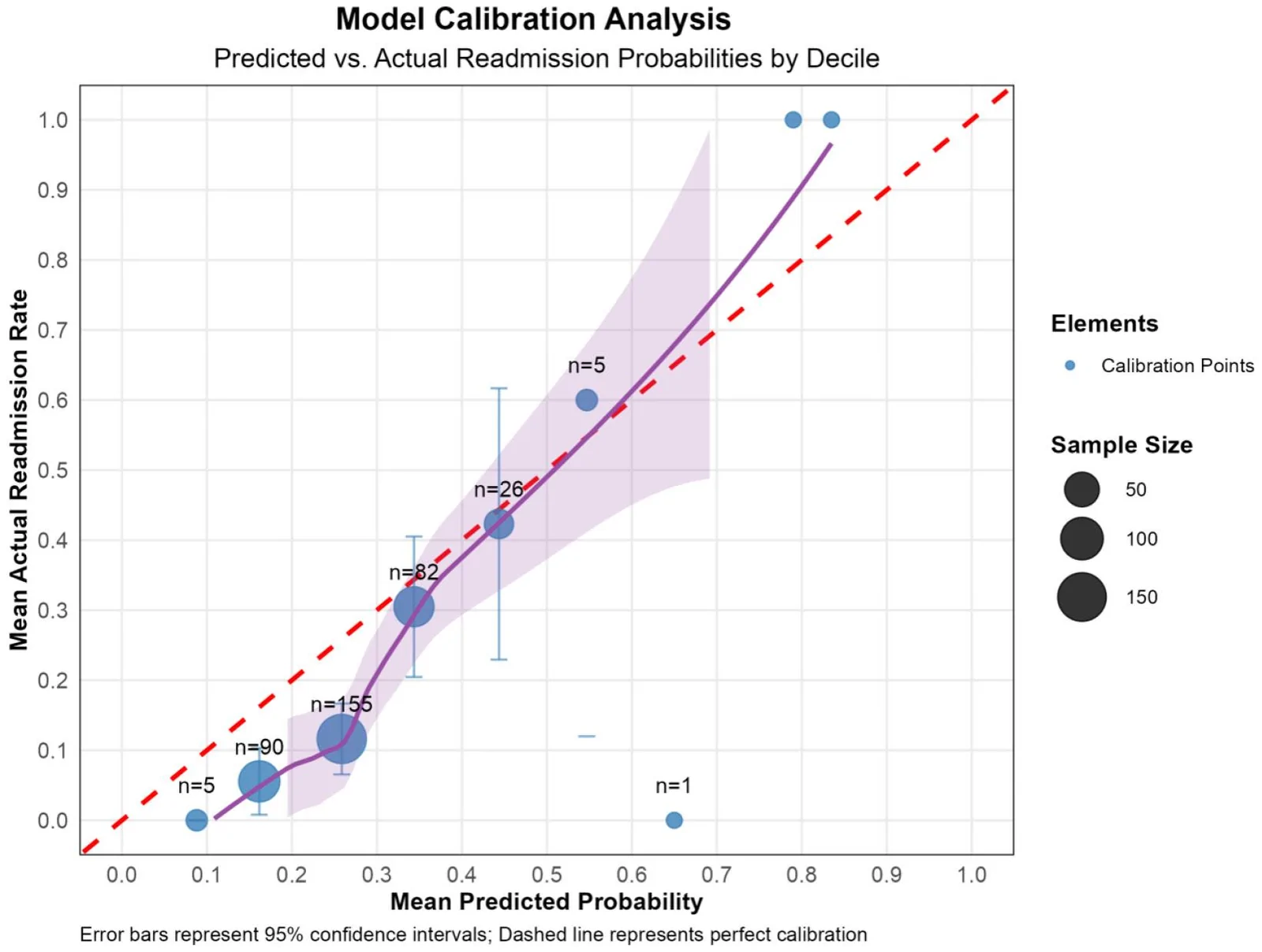
Model Calibration Analysis of the Tuned RF-SMOTE Model. *Note*. RE: Random forest; SMOTE: Synthetic minority over-sampling technique

## Discussion

 In this study, a tuned RF machine-learning model was developed and internally validated to predict one-year all-cause readmission in patients with STEMI who were undergoing PCI. The model achieved acceptable discrimination in handling class imbalance through SMOTE, resulting in a 17.6% readmission rate that corresponded to an AUC of 0.765, and categorizing patients into five risk groups with observed readmission rates ranging from 5.8% (very low-risk) to 100% (very high risk).

 Compared to logistic regression (AUC: 0.695), the RF model showed superior overall discriminative performance. However, the comparison revealed a distinct trade-off; LR achieved higher sensitivity (78.5%) at the cost of lower specificity (52%), implying that LR correctly identified more true positives but generated many false positives. Contrarily, RF reduced false positives at the expense of missing more true positives.

 Depending on the application, the RF model’s higher specificity may be advantageous when false positives are costly (e.g., unnecessary interventions or workups). Conversely, LR’s higher sensitivity might be preferred in screening contexts where missing a case carries greater risk.

 The superior performance of the RF model compared to conventional logistic regression aligns with the inherent capacity of ensemble methods to capture non-linear relationships and complex interactions among predictors without pre-specification. This finding corroborates with the results of previous comparative studies regarding cardiovascular risk prediction.^31,32^ However, logistic regression remains a valuable benchmark given its interpretability and established clinical utility.

 Likewise, the performance of RF model was consistent with that of previously developed STEMI-PCI readmission models, which have a range of AUC values from 0.72 to 0.80.^14,16,31,32^ A high NPV of 90.8% represented a significant strength, facilitating the identification of a low-risk group with a 9.2% likelihood of readmission, suitable for less intensive follow-ups in resource-constrained settings. This rule-out capability facilitates tiered interventions (e.g., telehealth and medication reviews for medium/high risk patients), thus optimizing resource allocation without compromising safety. In addition, the PPV (38.1%) indicated challenges in dealing with low-prevalence results, and the model’s detailed grouping enabled targeted, patient-focused care over simple diagnoses.

 The variable importance rankings revealed clinically significant predictors, with multivessel disease ranked the highest due to its correlation with procedural complexity and cardiac risk, followed by smoking, prolonged LOS, hypertension, partial thromboplastin time, EF, and age. These factors conform to the multifactorial causes of readmission, with multivessel disease and high blood pressure reflecting the top factors identified by Yao and Li.^14^ Additionally, the increased visibility of partial thromboplastin time signifies the post-STEMI hemostatic imbalance and extended LOS, indicating in-hospital complications, as supported by other studies.^14,19,33^

 Internal performance is being restricted by limitations that are preventing it from being generalized. Employing a single-center from a tertiary PCI registry may introduce selection bias, including the discrepancy between our 17.6% readmission rate and the national rate. Moreover, it restricts the generalizability of the findings; consequently, multicenter external validation is needed to evaluate calibration. In-hospital data retrospectively collected omitted post-discharge factor, including social determinants of health (SDOH), health literacy, and adherence. Tuning potential risk over fitting, which requires temporal validation and the future effect on readmissions, has yet to be proven. Consequently, missing SDOH data may underestimate disparities within underrepresented populations.

 External validation, the incorporation of SDOH through electronic health record (EHR) linkage, and prospective trials evaluating interventions guided by risk are crucial. In addition, the integration of EHRs via the use of SHapley Additive exPlanation values for real-time, explainable alerts could embed predictive models in workflows, transitioning from prediction to preventing readmissions.

**Highlights**

- The RF model predicted 1-year STEMI-PCI readmission (AUC: 0.765).
- The model identified several key risk factors, including multivessel disease, smoking, initial length of stay, and hypertension.
- High negative predictive value (90.8%) could effectively rule out readmission for low-risk patients.
- Patients were stratified into five risk groups, with readmission rates from 5.8% to 100%.

## Conclusion

 A clinically interpretable RF model was developed, which can effectively categorize patients with STEMI-PCI according to their one-year risk of readmission. The model identified a critical predictor (e.g., multivessel disease or smoking), and co-existing conditions provided a predictive tool that highlights areas for potential intervention efforts. Finally, further refinement and validation of this approach may lead to the development of personalized, resource-efficient post-discharge care pathways that are designed to decrease the number of unplanned hospital readmissions.

## Acknowledgments

 This study was supported by Vice-Chancellor of Research and Technology of Hamadan University of Medical Sciences (grant No. 140407296938). Accordingly, the authors would like to thank the clinical research development unit of Farshchian Heart Hospital, Hamadan University of Medical Sciences, Hamadan, Iran, for their assistance throughout the study period.

## Artificial Intelligence Use Disclosure

 AI tools were used solely for language editing and grammar improvement, rather than for data analysis, interpretation, or content generation. The authors assume full responsibility for the work.

## Competing Interests

 The authors declare that there is no conflict of interests regarding the publication of this paper.

## Ethical Approval

 This study was conducted in accordance with the ethical guidelines of the Declaration of Helsinki. Informed consent was waived since a retrospective design, and the study involved no direct interaction with the participants (only anonymized information was included). In addition, the data were completely de-identified, and no intervention was performed on patients. Finally, the study was approved by the Research Ethics Committee of Hamadan University of Medical Sciences (IR.UMSHA.REC.1404.669).

## Funding

 This study was financially supported by the Vice-Chancellor of Research and Technology of Hamadan University of Medical Sciences (grant No. 140407296938).
